# *Artemisia*, a Promising Tool for Integrated Parasite Control

**DOI:** 10.1155/japr/9987439

**Published:** 2025-07-01

**Authors:** Sharon Elizabeth Cruz-Estupiñan, Rosa Isabel Higuera-Piedrahita, Diana María Bulla-Castañeda, Javier Antonio Ballesteros-Ricaurte, Martin Orlando Pulido-Medellin

**Affiliations:** ^1^Grupo de Investigación en Medicina Veterinaria y Zootecnia (GIDIMEVETZ), Universidad Pedagógica y Tecnológica de Colombia, Tunja, Colombia; ^2^Facultad de Estudios Superiores Cuautitlán, Universidad Nacional Autónoma de México, Cuautitlán Izcalli, Mexico; ^3^Grupo de Investigación en Manejo de Información (GIMI), Universidad Pedagógica y Tecnológica de Colombia, Tunja, Colombia

**Keywords:** *Artemisia*, artemisinin gastrointestinal parasites, effect, resistance

## Abstract

The genus *Artemisia*, belonging to the family Asteraceae, comprises nearly 500 species with various pharmacological properties, such as antimalarial, antibacterial, antifungal, antioxidant, nematocidal, and cesticidal activities. Gastrointestinal parasites pose a significant health concern in animals, resulting in substantial financial losses due to the ineffectiveness of current prevention and control measures, further compounded by the rise of antiparasitic resistance. Consequently, there has been a surge in research endeavors aimed at identifying sustainable alternatives to address this issue, with a particular focus on herbalism due to its promise in this field. *Artemisia* has been identified as a source of secondary metabolites with the potential to kill parasites, making it a promising natural alternative to synthetic drugs. The objective of this article is to provide a comprehensive review of the genus *Artemisia* and its application in the control of gastrointestinal parasites. A comprehensive search was conducted using multiple databases, including Springer, Science Direct, Scopus, Web of Science, Latindex, PubMed, and SciELo, with specific keywords such as “*Artemisia*, Artemisin, gastrointestinal parasites, effect, resistance”. After a thorough review of the literature, 15 articles were identified as meeting the selection criteria. These articles encompassed studies on plants from the genus *Artemisia*, exploring their response to parasites in both free and endogenous life stages. Additionally, the review included studies on molecules derived from plants in the genus *Artemisia*, their ethnobotanical applications in addressing parasites, and their ethnoveterinary uses. These studies demonstrated that *Artemisia*, whether in vivo or in vitro, exhibited an impact on various gastrointestinal parasites and yielded positive or negative outcomes in the treatment of different parasite species.

## 1. Introduction

Gastrointestinal parasites are common in animals worldwide [1]. Infected animals often eat less or convert feed less efficiently [2]. These signs and behaviors are linked to changes in how proteins and energy are used, such as supporting the immune response to infection [3].

The increase in resistance to anthelmintics is a consequence of the heavy reliance on these broad-spectrum drugs [4]. There is a global distribution of drug resistance for the treatment of these parasitoses, and an increase in multiple resistance to these molecules has been reported worldwide. This represents an emerging problem in the treatment of parasitic diseases in both humans and animals [5–8]. It is also important to note that climate change has affected the geographic distribution and seasonal abundance of some parasites, as well as the interannual climate, which could lead to unexpected differences in the seasonal risk of parasitic infection between years [9].

In view of this problem, there is a trend to use bioactive compounds from different plants that have been shown to be cheap, efficient, readily available, and safe to use with minimal side effects in parasite control [10]. Currently, a considerable number of plants and their bioactive components with anthelmintic activity have been reported [11] since it has been shown that these compounds can have direct effects against populations of resident parasites in the gastrointestinal tract and can also improve the nutrition of the host as well as immunity against these parasites [12–14].

Within these plants is *Artemisia*, which has a unique position in modern traditional medicine and has several species, including *Artemisia cina* O. Berg ex Poljakov, *Artemisia annua* L., *Artemisia absinthium* L., *Artemisia asiatica* Nakai, *Artemisia douglasiana* Hook. & Arn., *Artemisia dracunculus* L., *Artemisia vulgaris* L., and *Artemisia brevifolia* Wall, have an important effect in the treatment of a variety of diseases, which have been used as potent antimalarials, anti-inflammatories, inducers of apoptosis, and antitumor [15], and their effect has been studied in the treatment of different diseases or parasitoses.

For instance, the anthelmintic properties of *Artemisia* species have been demonstrated in various studies. In India, *Artemisia* spp. showed a significant inhibition of egg hatching (88.3%) [16]. Similarly, in Pakistan, *A. brevifolia* reduced the number of eggs per gram (EPG) of feces by 67.2% in sheep with natural infections, 14 days posttreatment [17]. Additionally, *Artemisia maritima* L. and *Artemisia vestita* Wall. have shown a substantial decrease in EPG following the treatment period [18]. These findings underscore the necessity for a thorough review of the role of *A. cina* in the global control of gastrointestinal parasites.

## 2. Methodology

### 2.1. Search Strategy

The information search was carried out in the following databases: Springer, Science Direct, Scopus, Web of Science, Latindex, PubMed, and SciELo. The following terms were used as search criteria: “*Artemisia*, Artemisin, artemisinin, gastrointestinal parasites, *Artemisia*+nematicide, *Artemisia*+anthelmintic, *Artemisia*+parasites, *Artemisia*+*Haemonchus, Artemisia*+*Teladorsagia, Artemisia*+*Oesophagostomum, Artemisia*+*Trichostrongylus, Artemisia*+*Cooperia, Artemisia+Moniezia, Artemisia*+*Trichostrongylus, Artemisia*+*Fasciola*, effect, resistance”, for the search with appearance in the title, abstract, and keywords.

### 2.2. Inclusion and Exclusion Criteria

**Figure 1:**
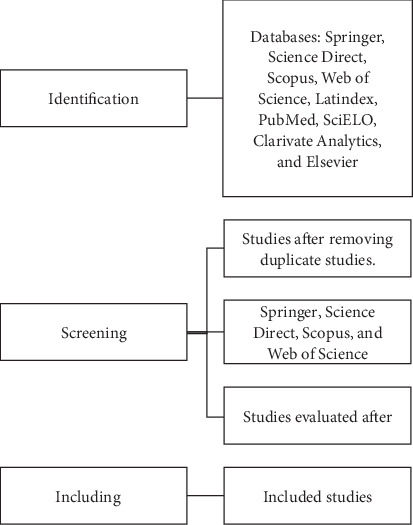
Flow diagram of the information selection process to carry out this review.

**Figure 2:**
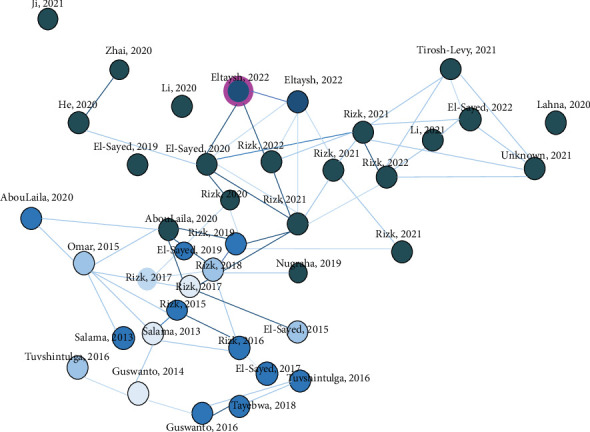
Author co-occurrence network map.

**Figure 3:**
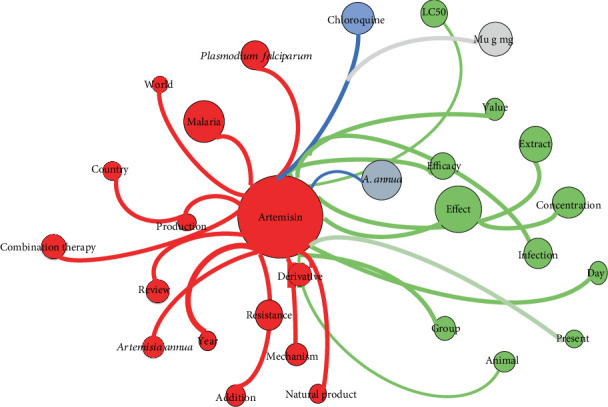
Keyword co-occurrence network map.

Brief communications, letters to the editor, books and book chapters, conference proceedings, and studies published up to 2024 were excluded from the analysis. Articles containing information on the use of *Artemisia* for the control of different gastrointestinal parasites were included, ensuring that all studies focused exclusively on animal use. The selection process is outlined in Figure 1, which presents a flow diagram of the information selection process for this review. After filtering the information, the database files and metadata were downloaded in RIS and BIB formats (Figures 2 and 3).

### 2.3. Data Analysis

Keywords and authors were the two main default units of analysis for the particular type of study. For coauthorship analysis, a maximum number of 10 authors per document and a minimum of one publication per author for a total of 14 authors displayed on the network map (Figure 2). Keywords were defined in a set of 115 articles. All analyses were performed with the VOSviewer Version 1.6 software using only Web of Science metadata which was where the greatest amount of information was found. The relationship maps were made with VOSviewer. The size of the bubbles in the maps represents the mean of the elements analyzed and the color the number of clusters where the data is grouped.

VOSviewer software analyzes and examines information, using scientific maps. His analytical approach is based on Jaccard index similarity measures and Pearson's correlation, where distances indicate the relationship and strength between the elements, that is, in graphical visualizations, where a smaller distance indicates a stronger relationship [19].

## 3. Results

The use of relationship maps in a literature review is a useful tool to identify and visualize the connections between the most relevant concepts and keywords in previous studies. In this case, the results of the literature review show that studies have been carried out with *A. annua* to treat malaria, suggesting that artemisinin, a compound present in the plant, may be effective in combating gastrointestinal parasites.

In the keyword map, “Artemisinin” is one of the most relevant keywords, indicating that it is an important research topic in this field. In addition, the medium-sized artemisinin-binding bubbles are related to concepts such as resistance, production, derivatives, and *Plasmodium*, suggesting that previous studies have investigated artemisinin resistance and the production of its derivatives, as well as its effectiveness against *Plasmodium*, the parasite that causes malaria (Figure 3).

In the blue bubble that binds artemisinin is *A. annua*, indicating that the plant has been extensively studied in relation to artemisinin and malaria. On the other hand, in the green bubble are the concepts of effect, efficacy, extract, and concentration, which suggests that previous studies have investigated the effects of *Artemisia* extracts and the concentrations of this plant on the efficacy against different diseases, among which are those generated by gastrointestinal parasites.

On the other hand, after the analysis of the 115 articles that provided the keywords, after the review, only 15 articles met the inclusion criteria, of which they were summarized in Table 1.

**Figure 4:**
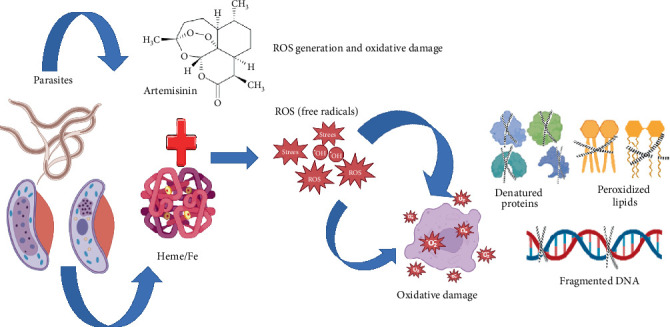
Reactive oxygen species generation and oxidative damage in parasite cells.

The most studied mechanism of *Artemisia* spp., specifically artemisinin, focuses on the generation of reactive oxygen species (ROS) (Figure 4). This process commences when artemisinin interacts with heme groups or ferrous iron (Fe^2+^) within the parasite, as observed in *Plasmodium* spp. (the causative agent of malaria). The cleavage of the peroxide bridge of the molecule releases free radicals (such as O_2_^−^ and H_2_O_2_) that oxidize proteins, lipids, and DNA, causing irreversible cellular damage and parasite death [34, 35]. Recent studies suggest that this mechanism could extend to nematodes like *Haemonchus contortus*, where oxidative stress alters antioxidant systems such as glutathione S-transferase [36].

**Figure 5:**
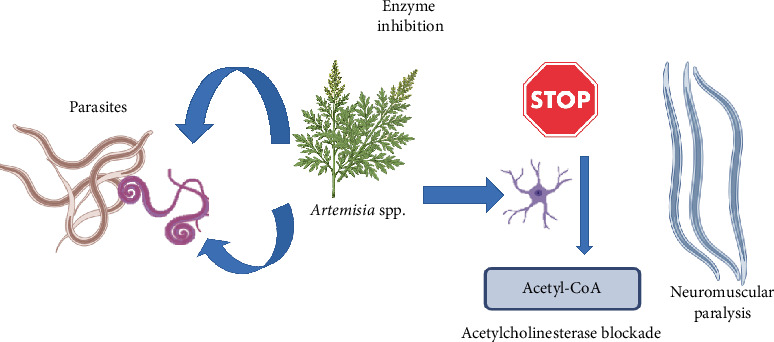
Enzyme inhibition leading to neuromuscular paralysis.

In addition to ROS, it has been proposed that *Artemisia* compounds exert enzyme inhibition (Figure 5), blocking key enzymes necessary for parasite survival. One such enzyme is acetylcholinesterase, which, when inhibited, leads to an accumulation of acetylcholine at neuromuscular synapses, resulting in paralysis and death in gastrointestinal nematodes [37]. While this mechanism is more commonly observed in other anthelmintics, it is hypothesized that derivatives of *Artemisia* could exhibit a similar effect, though further experimental evidence is required to substantiate this claim [37].

**Figure 6:**
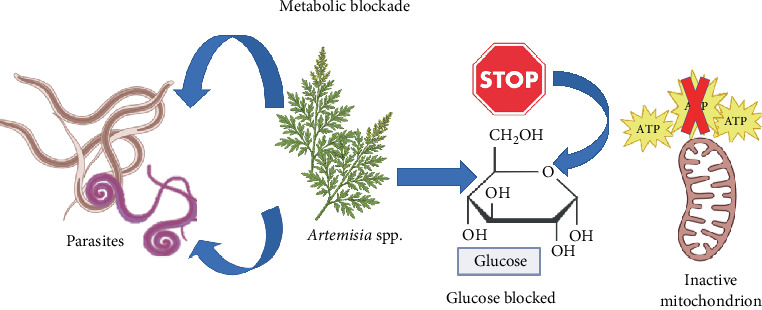
Metabolic blockade: inhibition of glucose uptake and ATP production.

Another critical axis is metabolic blockade (Figure 6), in which *Artemisia* disrupts essential energy pathways. By impeding glucose uptake and deactivating ATP production in mitochondria, the compounds deplete the parasite's energy reserves, leading to the paralysis of vital processes such as protein synthesis. This mechanism is pivotal in the context of *Schistosoma* spp., where it modifies glycogen metabolism [38], and in the case of *Plasmodium* spp., by compromising the mitochondrial respiratory chain [34, 35].

Among the complementary mechanisms, immunomodulation stands out, where *Artemisia* derivatives enhance the host response by stimulating T cells and proinflammatory cytokines [38], as well as interference with macromolecule synthesis, such as the inhibition of DNA replication in *Plasmodium* [34, 35]. However, parasites like *Plasmodium falciparum* have developed resistance through adaptive strategies such as reducing metabolic activity (quiescence) and modifications in tRNA to avoid ROS activation [39].

The precise mechanisms by which *Artemisia* exerts its anthelmintic effects on gastrointestinal parasites, such as *Haemonchus*, remain to be fully elucidated. However, several potential mechanisms have been postulated, including oxidative stress (Figure 4), *β*-tubulin inhibition (a property shared with other anthelmintics), or alteration of antioxidant pathways [36, 37]. The versatility of these compounds underscores their therapeutic potential; however, emerging resistance necessitates the exploration of new targets, such as epitranscriptomic modifications or gene expression regulation, to design more effective and sustainable therapies.

## 4. Discussion

Different studies have demonstrated the anthelmintic effect of *Artemisia* spp., for example, the one developed in India, where a significant inhibition (88.3%) of egg hatching was found [16]. In this work, crude extracts of the aerial parts of *A. absinthium* showed significant anthelmintic effects during the adult immersion test and the in vivo test with a fecal egg count (FEC) of 90.46% in sheep (administered with 2.0 g/kg body weight) 15 days after treatment. It was concluded that *A. absinthium* extracts are a promising alternative to commercially available anthelmintics for the treatment of gastrointestinal nematodes in sheep [16].

The effect of *A. brevifolia* (whole plant) on live *H. contortus* was revealed in Pakistan—there was mortality within 6 h of exposure. The whole plant was administered as a crude powder at 3 g/kg body weight to sheep, and there was a reduction (67.2%) of EPG of feces 14 days after treatment [17].

On the other hand, Irum et al. [18] found a significant decrease in FEC after the posttreatment period for *A. maritima* and *A. vestita.* The greatest reduction in FEC for *A. vestita* was 87.2% at 100 mg/kg, whereas for *A. maritima*, it was 84.5% on Day 28 posttreatment. Zhu et al. [20] in *Artemisia lancea* concluded that this plant had some degree of anthelmintic activity against *H. contortus* eggs and larvae with more than 99% inhibition of egg hatching.

In Pakistan, it was observed that *Artemisia sieversiana* and *Artemisia parviflora* after 27 h had a very significant ability to inhibit egg hatching (100%); it was recorded for both plant extracts, while the highest activity for the worm assay adults and larvicide of the trial was 90% for *A. sieversiana*. The highest activity for adult motility and larvicidal assay for *A. parviflora* was 89% and 86.6%, respectively [21]. In *Artemisia absinthium*, the results were very similar in Europe, specifically in Slovakia, where Váradyová et al. [22] demonstrated how the methanolic extracts of *A. absinthium*, in comparison with other plants, presented the most significant anthelmintic effects (*p* < 0.05). In another study in Africa (Tunisia) that was carried out with the ethanolic extract of *Artemisia campestris*, after 8 and 24 h of exposure, it produced 91.3% and 100% mortality at the highest concentration tested, respectively, while the aqueous extract induced 3.22% and 100% mortality, with 70.96 % at the same concentration, respectively [23]. The results obtained in previous investigations show that in vitro experiments are useful to find favorable chemotherapeutic alternatives for the development of new antiparasitic agents.

On the other hand, the effect of *A. cina* has been estimated in different larval stages, in transitional larvae (third-stage (L3)–L4) (HcTrL3–L4) of gerbils artificially infected with *H. contortus* (HcArt/inf/gerbs). Ethyl acetate (Ac-EtOAcEx), n-hexane (Ac-n-HexEx), and methanol (Ac-MethEx) extracts of *A. cina* were evaluated at 1 and 2 mg/mL against HCL3 and HcTrL3–L4 to 24 h of exposure. The highest in vitro activities (75% and 82.6%) were shown by Ac-n-HexEx at 1 and 2 mg/mL, respectively. For HcTrL3–L4, the highest in vitro activities (69% and 23%) were shown by Ac-n-HexEx and isoguaiacine at 0.625 mg/mL, respectively [24].

The anthelmintic activity has been compared both in vivo and in vitro, for example, Cala et al. [25] used crude extracts of *A. annua* in vitro and compared the most effective extract with artemisinin in sheep naturally infected with *H. contortus*. They were evaluated in vitro by the egg hatch test (EHT) and with the bicarbonate extract alone for the larval development test (LDT) using *H. contortus.* Sheep treated with artemisinin and *A. annua* had nonsignificant HPG reductions of 28% and 19%, respectively, while sheep in the infected/untreated group had an average egg per gram increase of 95%. Sheep treated with artemisinin and *A. annua* maintained blood hematocrit throughout the experiment, while untreated/infected controls had a significant reduction in hematocrit.

In another work, in Mexico, the toxicity of *A. cina* 30 CH as a homeopathic product was compared against *Haemonchus contortus* in in vitro and in vivo tests where the toxicity of *A. cina* 30 CH against *H. contortus* was observed in infected lambs. Seven days after infection, in addition to a 69% reduction in the mean number of EPG at 28 days posttreatment, similar to albendazole (*p* < 0.05), which agrees with the work carried out by Higuera-Piedrahita et al. [26] with Ac-n-HexEx as a natural anthelmintic treatment for peripartum goats naturally infected with the nematodes *H. contortus* and *Teladorsagia circumcincta*, parameters such as FEC, ocular mucosa color (OMC), and body condition (BC). The FEC, OMC, and BC parameters were recorded at 0 days (peripartum period), 7 days (birth period), and 23 days (postpartum). On Day 23 (*p* < 0.05) of the experiment, significant differences were observed in OMC or BC compared to the control group, for BC in the group treated with *A. cina* [26].

However, another study indicated that the use of medicinal plants such as *A. absinthium* showed no significant in vivo effects in lambs infected with *H. contortus*. In a similar vein, fractions obtained from *A. cina* were observed to have no potential effect on the L3 association [40].

In contrast, recent studies in Mexico have identified specific compounds with activity against *H. contortus*. Notably, cynic acid, a novel sesquiterpene obtained from *A. cina,* was identified as a significant anthelmintic agent against L3 larvae of *H. contortus*, suggesting its potential as an antiparasitic agent [32].

This study identified pharmacodynamic synergies between key compounds, including peruvin, 1-nonacosanol/hentriacontane, and cynic acid itself. These findings elucidate the underlying mechanisms by which formulations derived from crude extracts, as previously examined [33], might exhibit inadequate efficacy due to chemical antagonism or suboptimal concentrations of active metabolites. Consequently, the strategic design of mixtures emerges as a novel approach to enhance the anthelmintic effect.

Other investigations have also demonstrated the effect of *Artemisia* sp. against other parasites; the aqueous extract of *Artemisia sieberi* showed to have protoscolicidal activity in Iran and can be considered a natural agent against the protoscolices of the hydatid cyst of *Echinococcus granulosus* [27]. The results indicated that the plant extract is effective at all concentrations tested against cestodes such as *Moniezia expansa, M. denticulata, M. benedeni, M. trigonophora*, and *Thysaniezia giardi*, and *T. giardi*. This suggests that the aqueous extract of *A. sieberi* has an antiparasitic effect against *E. granulosus*. On the other hand, it was indicated that more clinical and in vitro studies are required to investigate the effect of different extracts of this plant and its components as a natural scolicidal agent. Most studies showed an effect at different stages of the parasites, making different species a viable treatment option for GI parasites.

Despite the paucity of elucidated mechanisms of action of *Artemisia* spp. in gastrointestinal parasites, the in vitro [21] and in vivo [16] efficacy of this plant supports its potential. However, the in vivo results obtained from studies [16, 40] are contradictory, underscoring the necessity for further investigation into specific mechanisms that extend beyond empirical observations. While the role of ROS generation in *Plasmodium* is well-established [34, 35], a more nuanced relationship with nematodes is postulated [36], though this remains to be substantiated at the molecular level. It is imperative to identify precise targets, such as enzymes and *β*-tubulin [36, 37], as well as resistance strategies like quiescence [39]. Recent findings, such as the synergy between compounds [32], underscore the significance of optimized formulations [33] to overcome antagonism in crude extracts. The versatility of *Artemisia*, including its efficacy against cestodes [26], underscores its potential as a natural alternative. However, its success is contingent upon the integration of traditional herbal practices with rigorous pharmacological validation, a necessity in the context of escalating resistance to conventional treatments.

## 5. Conclusion

A review of the extant studies indicates the potential of *Artemisia* for the control of gastrointestinal parasites. However, further research is necessary to identify the specific compounds responsible for its antiparasitic effects and to determine which *Artemisia* spp. are most effective against different parasite groups, such as nematodes and cestodes. This focus is imperative in light of the contemporary transition toward integrated parasite control and environmentally sustainable production systems. Additionally, our comprehension of the molecular mechanisms underlying these effects remains limited. Future investigations should aim to elucidate the precise molecular interactions and structural characteristics of the bioactive compounds, which will be critical for optimizing natural treatment strategies and overcoming resistance to conventional therapies.

## Figures and Tables

**Table 1:** Summary of selected studies on the antiparasitic effects of *Artemisia* species.

***Artemisia* species**	**Effect**	**Country**	**Author**	**Databases**
*Artemisia lancea*	Greater than 99% inhibition of hatching of *Haemonchus contortus* eggs [20]	China	Zhu et al. [20]	Science Direct/PubMed/Scopus/Web of Science
*Artemisia sieversiana* and *Artemisia parviflora*	27 h inhibition of egg hatching (100%), larvicidal activity of the assay was 90% for *A. sieversiana; A. parviflora* was 89% [21]	Pakistan	Váradyová et al. [21]	Science Direct/PubMed/Scopus/Web of Science
*Artemisia maritima* and *Artemisia vestita*	Fecal egg count reduction for *A. vestita* was 87.2% at 100 mg/kg, while for *A. maritima*, it was 84.5% on Day 28 after treatment [18]	Pakistan	Irum et al. [18]	Science Direct/PubMed/Scopus/Web of Science
*Artemisia absinthium*	The methanolic extracts in comparison with other plants, presented the most significant anthelmintic effects (*p* < 0.05).	Slovakia	Váradyová et al. [22]	Science Direct
*Artemisia campestris*	After 8 and 24 h of exposure, it produced 91.3% and 100% mortality in adults, *Haemonchus contortus*	Tunisia	Akkari et al. [23]	Springer/Scopus
*Artemisia cina*	*A. cina* extracts: at 1 and 2 mg/mL vs. 4 after 24 h of exposure. The highest in vitro activities (75% and 82.6%), at 2 mg/mL, (69% and 23%) were shown by Ac-n-HexEx and isoguvacine at 0.625 mg/mL, respectively.	Mexico	Higuera-Piedrahita et al. [24]	Springer
*Artemisia annua*	In sheep hematocrit maintenance provided by artemisinin and the extract, there were nonsignificant EPG reductions of 28% and 19%.	Brazil	Cala et al. [25]	Springer
*Artemisia cina*	EPG reductions were recorded in the h-Acn treated group as follows: 20.1% ± 34.4% and 31.7% ± 38.2% on Days 7 and 23 compared to the control group.	Mexico	Higuera-Piedrahita et al. [26]	Springer
*Artemisia sieberi*	The present study suggests that the aqueous extract of *A. sieberi* has an antiparasitic effect against *Echinococcus granulosus.*	Iran	Vakili et al. [27]	Science Direct/PubMed
*Artemisia spicigera*	The results revealed that the removal of ocysts was significantly reduced (*p* < 0.05) in the treatment groups. There was no significant difference between the mean weight gain/loss in the control and treated groups.	Iran	Shahbazi et al. [28]	Scopus/PubMed
*Artemisa absinthium*	Despite large individual differences among treated lambs in egg reduction, the mean eggs per gram (EPG) of *Haemonchus contortus* between the treated and untreated groups did not differ significantly (*p* > 0.05).	Slovakia	Mravčáková et al. [29]	Scopus/PubMed
*Artemisia annua*	In vitro tests show that the *A. annua* extract used has the potential to be a good drug candidate against *H. contortus.*	Brazil	Sprenger et al. [30]	Scopus
*Artemisia absinthium*	Strong ex vivo activity was shown against the L1 larvae of the nematode *Trichinella spiralis* with a reduction in infectivity between 72% and 100% in a dose range of 0.5–1 mg/mL in the absence of cytotoxicity against mammalian cells. In addition, the in vivo activity of against *T. spiralis* showed a 66% reduction in intestinal adults.	Spain	García-Rodríguez et al. [31]	Scopus
*Artemisia cina*	The efficacy of n-hexane, ethyl acetate, and methanol extracts was assessed against third-instar larvae of *Haemonchus contortus*. The ethyl acetate extract exhibited an LC50 of 2.56 mg/mL and an LC90 of 3.30 mg/mL. The freshly isolated sesquiterpene (cynic acid) demonstrated an LC50 of 0.13 mg/mL and an LC90 of 0.40 mg/mL, exhibiting a 256-fold increase in activity at the LC50 and a 15.71-fold increase at the LC90 compared to the ethyl acetate extract.	Mexico	Arango-De la Pava et al. [32]	PubMed
*Artemisia cina*	The ethyl acetate extract of *A. cina* contained four bioactive compounds: 1-nonacosanol, hentriacontane, peruvin (sesquiterpene lactone), and cynic acid (sesquiterpene). Of these, cynic acid demonstrated the highest lethality in L3 larvae of *Haemonchus contortus.* (LC50: 0.154 mg/mL), while showing no effect on eggs. Peruvin exhibited larvicidal and hatching inhibition activity, while the 1-nonacosanol/hentriacontane mixture effectively blocked hatching. The effects of these mixtures were further enhanced by synergistic binary combinations.	Mexico	Arango-De la Pava et al. [33]	PubMed

## Data Availability

Data sharing is not applicable to this article, as no new data were created or analyzed in this study.
